# Reaction wood – a key cause of variation in cell wall recalcitrance in willow

**DOI:** 10.1186/1754-6834-5-83

**Published:** 2012-11-22

**Authors:** Nicholas JB Brereton, Michael J Ray, Ian Shield, Peter Martin, Angela Karp, Richard J Murphy

**Affiliations:** 1 Department of Life Sciences, Imperial College, London, SW7 2AZ, UK; 2 Rothamsted Research, Harpenden, AL5 2JQ, UK; 3 The Agronomy Institute, Orkney College, University of the Highlands and Islands, East Road, Kirkwall, Orkney, KW15 1LX, UK

**Keywords:** Biofuel, Willow (*Salix*), Lignocellulose, Reaction wood, Recalcitrance (saccharification), Cell wall Composition

## Abstract

**Background:**

The recalcitrance of lignocellulosic cell wall biomass to deconstruction varies greatly in angiosperms, yet the source of this variation remains unclear. Here, in eight genotypes of short rotation coppice willow (*Salix* sp.) variability of the reaction wood (RW) response and the impact of this variation on cell wall recalcitrance to enzymatic saccharification was considered.

**Results:**

A pot trial was designed to test if the ‘RW response’ varies between willow genotypes and contributes to the differences observed in cell wall recalcitrance to enzymatic saccharification in field-grown trees. Biomass composition was measured via wet chemistry and used with glucose release yields from enzymatic saccharification to determine cell wall recalcitrance. The levels of glucose release found for pot-grown control trees showed no significant correlation with glucose release from mature field-grown trees. However, when a RW phenotype was induced in pot-grown trees, glucose release was strongly correlated with that for mature field-grown trees. Field studies revealed a 5-fold increase in glucose release from a genotype grown at a site exposed to high wind speeds (a potentially high RW inducing environment) when compared with the same genotype grown at a more sheltered site.

**Conclusions:**

Our findings provide evidence for a new concept concerning variation in the recalcitrance to enzymatic hydrolysis of the stem biomass of different, field-grown willow genotypes (and potentially other angiosperms). Specifically, that genotypic differences in the ability to produce a response to RW inducing conditions (a ‘RW response’) indicate that this RW response is a primary determinant of the variation observed in cell wall glucan accessibility. The identification of the importance of this RW response trait in willows, is likely to be valuable in selective breeding strategies in willow (and other angiosperm) biofuel crops and, with further work to dissect the nature of RW variation, could provide novel targets for genetic modification for improved biofuel feedstocks.

## Introduction

Producing liquid biofuels from lignocellulosic plant biomass has the potential to contribute to global carbon mitigation targets, improve rural regeneration and increase energy security [1-3]. Dedicated bioenergy crops, such as Short Rotation Coppice (SRC) willow (*Salix* sp.) and poplar (*Populus* spp.) (which share genomic macrosynteny [4]), are considered to play a vital role in future sustainable production of lignocellulose derived liquid biofuels due to their potential for high biomass yields with low agricultural inputs in long-term perennial cropping systems [5-8]. Moreover low-input, dedicated bioenergy crops like willow do not require the same quality of land that is necessary for food production [9], thereby potentially unlocking land where options are limited for cultivation and minimising conflict between food and energy needs. Whilst enhancing the biomass yield per unit area of land is an essential target for improvement of these dedicated bioenergy crops, the quality of the biomass and the ease with which it can be converted downstream into liquid biofuels deserves equal, if not more, attention. This is because biomass quality not only influences the amount of energy/fuel that can be obtained from a given land area but also affects the unit costs and environmental footprint of the fuel produced. The main polymeric components of lignocellulosic plant cell walls (cellulose, lignin and hemicelluloses) form a resilient complex that is resistant to deconstruction (recalcitrance). A considerable proportion of the energy required to process lignocellulosic biomass to liquid biofuels is therefore expended in pretreatment steps designed to overcome this recalcitrance to deconstruction [10-12]. Much research in this area is currently focused on identifying optimised pretreatment systems, in which feedstocks are matched with the most appropriate pretreatment method as well as, more fundamentally, attempting to link their cell wall characteristics with their cell wall recalcitrance.

**Figure 1:**
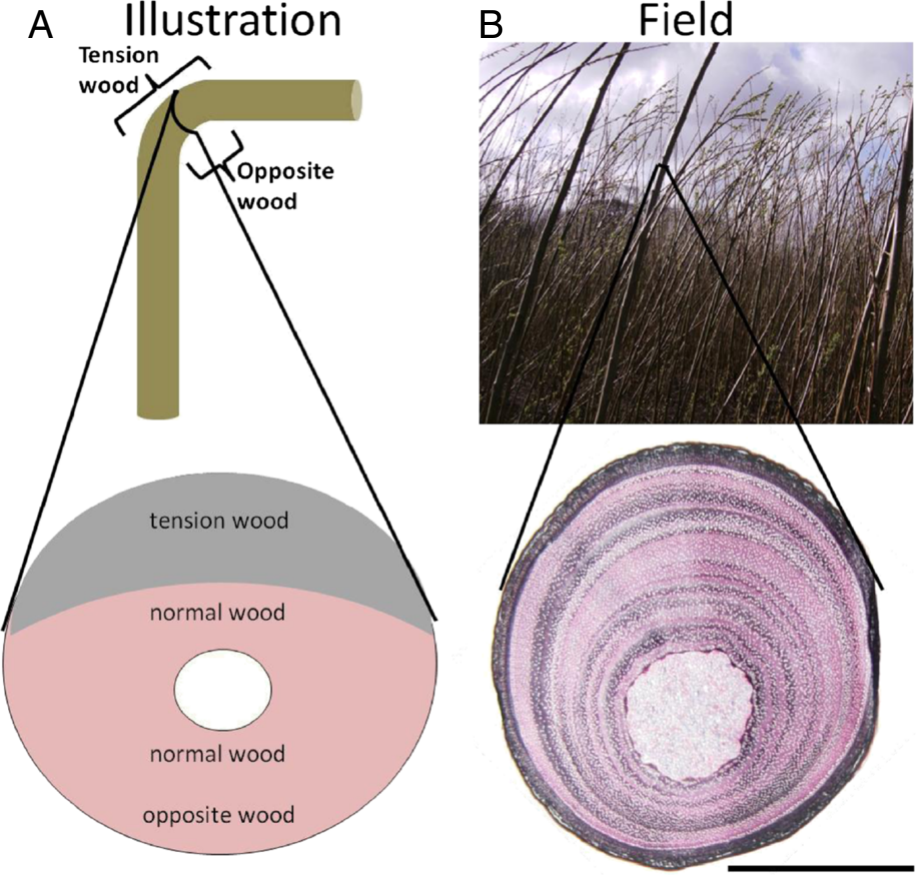
**A Illustrations depicting the traditional notion of reaction wood. Top**: a single stem bent away from the vertical, **Bottom**: a transverse section showing the tension wood region more darkly shaded. **B** Images displaying reaction wood in field-grown willow. **Top**: Photograph of mature willow stems grown in a UK field trial. **Bottom**: Midpoint 20-μm transverse section of a single stem from a mature field-grown willow tree. Stained in 1% Chlorazol Black E in methoxyethanol (black – binds specifically to the gelatinous layer within the G-fibres of tension wood [21]) and 1% aqueous Safranin O (red – binds to the secondary cell wall in a non-specific manner). Scale bar = 5 mm.

Reaction Wood (RW) formation is an innate physiological response by woody plants to counteract environmental stimuli, either thigmomorphogenic (mechanical stress) or gravitropic (gravitational perception) in nature [13,14], by structurally reinforcing the plant and redirecting growth towards the vertical. RW is thus commonly thought to be found predominantly in branch wood and in leaning stems. However, it is seen also in vertical stems, where it has been suggested that RW can form in response to internal growth strains resulting from rapid growth [15]. Woody gymnosperms form a type of RW termed *compression wood* which occurs on the ‘lower’ (compression) side of the stem or branch. In woody angiosperms, such as willow and poplar, RW comprises *Tension Wood* (TW) which is formed on the ‘upper’ (tension) side of the stem or branch and *Opposite Wood* (OW), a polarised antagonistic response formed on the ‘lower’ side of the stem or branch (Figure 1A). Tension wood is often characterised by the formation of a gelatinous layer within the fibre cells (G-fibres) of the secondary xylem. This unique cell wall layer differs from the normal fibre cell wall and is thought to be non-lignified and mainly composed of cellulose with the potential additions of arabinogalactan and xyloglucan [16-18]. Less is known regarding OW composition in angiosperms and only recently has it been shown to have the defining characteristic of increased lignin and cell wall recalcitrance when compared with normal wood [19,20].

Previous studies have recognised that general cell wall composition and recalcitrance to enzymatic saccharification in both willow and poplar exhibit genotype-specific, natural variation [22-26]. Surprisingly, whilst extreme transgenic low-lignin phenotypes (e.g. < 15% lignin on a mass basis) show reduced recalcitrance [27], none of the natural variation in basic cell wall compositional components (such as lignin and sugar contents) account sufficiently well for this variability in cell wall recalcitrance, leaving its fundamental causes unresolved. A number of studies have characterised the composition of the cell walls of RW (TW & OW) and normal wood (NW) as well as their response to pretreatment and/or enzymatic saccharification [19,23,28,29]. There is compelling evidence from this literature that ‘isolated’ TW has cell wall sugars that are more accessible to enzymatic saccharification when compared with NW and/or OW and, importantly, that RW induction can influence net cell wall recalcitrance over the ‘whole tree’ biomass. For the present research the entire ground stem biomass was assessed in order to observe the net impact of RW induction at the whole tree level. Previous studies have focused on comparisons of TW and OW in individual trees whereas the current research utilises multiple trees in order to draw conclusions regarding genotypic variation. There have been no reports to date indicating whether there is variation in the ability to form RW among genotypes and, if so, whether variation in responsiveness to such conditions can contribute to genotype-specific variation in cell wall recalcitrance. Quantification of the proportions of the individual components of RW (TW, OW) and NW in whole tree stem biomass in the field is not possible as no comprehensive and unambiguous techniques currently exist (Figure 1B). The amount of G-fibres can be visualised using histology on single transverse sections of wood [21], but this gives little indication of their mass proportion over the whole length of the stem/tree. Also, the amount of OW, which recent phenotypic and transcriptomic work indicates is distinct from normal wood [19,30-32], cannot easily be distinguished based on histology. Because of the above, we have been careful in this work to focus our experimentation and interpretation on exploring the potential effects in terms of overall RW and to avoid unsupported linkages to TW formation.

Here we aim to address two main questions regarding the effects of RW inducing conditions on the recalcitrance of SRC willow stem biomass:-

1) Do genotypic differences occur in enzymatic glucose release at the whole tree level in response to controlled RW inducing conditions? Such differences can be used to indicate a RW response in the material examined. A pot experiment was devised to test whether variation exists in the enzymatic glucose release from eight genotypes of willow. The results from this were compared with enzymatic glucose release from mature, field grown trees of the same genotypes.

RW response was then explored further in field-grown trees to address the second question:-

2) Do higher RW inducing field conditions impact on cell wall recalcitrance of mature trees? To address this samples were taken from a field trial at Orkney (UK), where the willows were exposed to potentially high RW inducing conditions (long durations of wind and high maximum wind-speeds).

## Results

### The influence of reaction wood induction on cell wall properties in the pot trial

**Figure 2:**
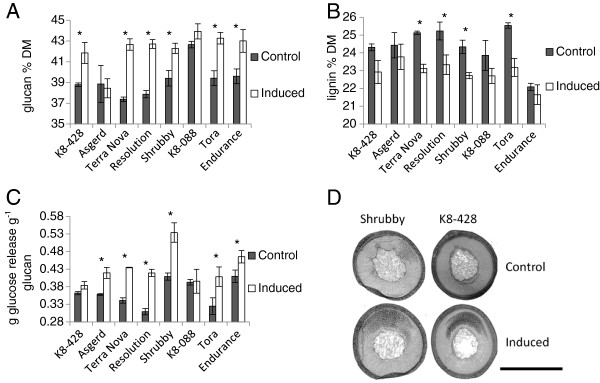
**Control and Reaction Wood induced potgrown trees of eight genotypes.A** Glucan composition expressed as a percentage of dry matter (DM). **B** Lignin composition expressed as a percentage of DM. **C** Glucose yields from enzymatic saccharification presented as grams of glucose released per gram of glucan present in the biomass. Error bars represent standard error (n = 3 trees). Full mass closed compositional tables are available in supplementary information. **D** Midpoint 20-μm transverse sections of a single stem from pot-grown genotypes ‘Shrubby’ and ‘K8-428’. Stained in 1% Chlorazol Black E in methoxyethanol (black – binds specifically to the gelatinous layer within the G-fibres of tension wood [21]) Scale bar = 5 mm. * Significant difference (*t*-test, p < 0.05).

The pot trial was designed to assess what influence RW induction (by growing the trees at a 45° angle to the vertical) would have on willow cell wall composition and cell wall sugar accessibility. Glucan accessibility, measured by enzymatic saccharification (cell wall recalcitrance), and cell wall composition of the whole stem biomass were significantly altered by the induction of RW in almost all genotypes (Figure 2). With respect to composition, the exceptions were the genotypes ‘Asgerd’ and ‘K8-088’ which *did not* have significantly (*t*-test, p > 0.05) altered glucan or lignin content upon RW induction. The genotypes, ‘K8-428’ and ‘Endurance’ *did* have significant differences in glucan content but *not* significantly altered lignin content upon RW induction (Figure 2A and B). Glucose release, expressed as a proportion of the glucan within the cell wall, signifies how *accessible* this glucose is to enzymatic saccharification. A broad range of glucan accessibility was seen between the genotypes, ranging from 0.30 to 0.53 g of glucose per gram of glucan. The disparity between these values reflects the impact of RW induction on the genotypes, with all except ‘K8-428’ and ‘K8-088’ showing significantly altered cell wall accessibility (Figure 2C). Transverse sections were made from two of the genotypes for histological analysis, ‘Shrubby’ and ‘K8-428’, representing the extremes for alteration in glucan accessibility upon RW induction (ie highly increased and no significant change, respectively) (Figure 2D). The two genotypes could not be distinguished on the basis of the observed abundance of G-fibres. It has been accepted convention that assessment of tension wood (as abundance of G-fibres) can be used to indicate the extent of RW response for angiosperms, but the present findings suggest that it is difficult to use their abundance as an accurate reflection of the entirety of RW response in these trees.

Variation in glucan content and variation in glucan accessibility both contribute to the final glucose yield of a feedstock, which is strongly indicative of final ethanol yields. Substantial ranges in glucose yield, from 0.12 to 0.23 g of glucose per gram of Dry Matter (DM), resulted from these different genotypes and conditions. The genotype ‘K8-088’ (which did not have significantly different glucan content) *did not* have significantly altered final glucose yields after RW induction whereas the genotype ‘K8-428’, although *not* showing an increase in glucan *accessibility*, *did* have increased glucan *content*, which resulted in a significantly increased final glucose yield. Overall biomass yields *did not* differ significantly between control and RW induced trees for any genotype, although they did vary between genotypes (Additional file 1: Table S1).

### The relationship between juvenile pot-grown phenotype and mature field-grown phenotype

**Figure 3:**
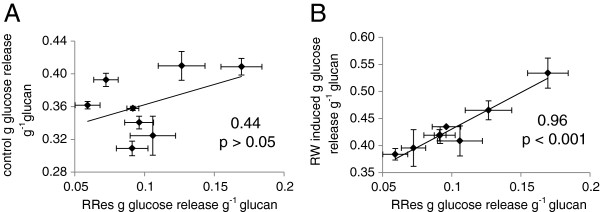
**Correlations of glucose yields from enzymatic saccharification for eight*Salix*genotypes.** Glucan accessibility from mature field-grown (Rothamsted Research site – RRes) trees correlated against glucan accessibility from: **A** control pot-grown trees and **B** reaction wood induced pot-grown trees. Glucan accessibility expressed as grams of glucose release per gram of glucan present in the biomass. Correlation coefficients and significance level displayed. Error bars represent standard error (n = 3 trees).

As the genotypes showed clear variation in the response to RW induction in the pot trial the saccharification and compositional data for these juvenile trees were compared with those from mature trees of the same genotypes grown in a field trial at Rothamsted Research (RRes) and assessed at the end of a three year harvest cycle (with seven year-old root stocks) [24]. ANOVA was performed on data sets prior to correlation coefficients being assessed, all glucose release yields used in the correlations showed significant differences (ANOVA, p < 0.01). No significant correlation (p > 0.05) was found between glucose release (per gram of glucan) from control pot-grown willows and glucose release of mature field-grown trees (Figure 3A). However, glucose release from the RW induced pot-grown trees showed a very strong and significant correlation with that of the mature field-grown trees, having a correlation coefficient of 0.96 (p < 0.001) (Figure 3B).

### Impact of potentially higher reaction wood inducing conditions on mature field-grown phenotype

The substantial differences observed in glucose release yields between control and RW induced pot-grown trees in some genotypes led us to hypothesise that a field environment with potentially higher RW inducing conditions (e.g. long durations of wind and high maximum wind-speeds) could lead to trees with higher glucose release yields. An opportunity to examine this was provided by the fact that a number of genotypes present in the RRes field trial [24] were also cultivated in a similar trial at a site on Orkney, UK, where trees are exposed to long periods of windy weather and high maximum wind-speeds due to north Atlantic weather systems. Between January 2008 and December 2010 the average wind speed was 6.36 (sd 2.65) meters per second at a height of two meters in Kirkwall (Orkney) and 2.60 (sd 0.99) at the same height in RRes (Weather data from UK Meteorological Office ARCMET and TELEX databases).

**Figure 4:**
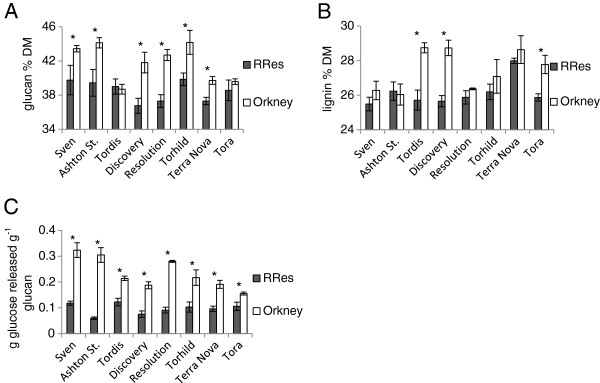
**Mature field**-**grown trees of eight genotypes grown at the Rothamsted Research** (**RRes**) **and Orkney sites.A** Glucan composition expressed as a percentage of dry matter (DM). **B** Lignin composition expressed as a percentage of DM. **C** Glucose yields from enzymatic saccharification presented as grams of glucose release per gram of glucan present in the biomass. Error bars represent standard error (n = 3 trees). Full mass closed compositional tables are available in supplementary information. * Significant difference (*t*-test, p < 0.05).

At the Orkney site (representing a higher RW inducing environment) the cell wall composition was significantly altered in most genotypes, to differing degrees, when compared with the same genotypes grown at the RRes site (representing a lower RW inducing environment) (Figure 4A and B). ‘Resolution’, as in the pot trial, had substantially increased glucan content under the higher RW inducing conditions at Orkney. Only ‘Tordis’ and ‘Tora’, *did not* have significantly altered glucan content (*t*-test, p > 0.05) and only ‘Tordis’, ‘Tora’ and ‘Discovery’ *did* have significantly altered lignin content (*t*-test, p < 0.05).

More striking than the shifts in composition were the substantial changes in cell wall accessibility. All the genotypes had increased glucan accessibility but, again, increases were highly varied and genotype-specific. The genotypes ‘Tora’ and ‘Tordis’ had the smallest increases in accessibility of approximately 45% and 75% more glucose released per gram of glucan from material grown at Orkney. The greatest change in glucan accessibility was seen in ‘Ashton Stott’ where trees grown in Orkney had a five-fold increase in glucose released per gram of glucan compared with trees grown at RRes.

## Discussion

### The influence of reaction wood induction on cell wall properties within the pot trial

A novel strategy adopted in this work was to induce RW in a set of previously characterised genotypes [24] in a consistent manner under controlled conditions and then to assess cell wall composition and glucan accessibility. Whilst this approach does not yield information regarding the local polarised effects of RW formation (direct variation in the *amount* of TW and/or the *amount* of OW of a part of a single stem) it does avoid the inherent problems associated with estimating the relative proportions of these different tissues throughout an entire stem. Because of this approach, and due to the fact that pot-grown biomass yields did not vary with RW induction in any of the genotypes, any improvements in sugar release yields should translate to real downstream yield benefits.

With the exception of two genotypes (which did not change significantly), a general trend was found of RW induction resulting in increased glucan content. This has been well documented from the first studies into RW and is often related to an increased number of G-fibres [28,33]. What is more interesting though is that these six genotypes showed differing degrees of increase in glucan content, demonstrating genotype-specific variation in the type and/or degree of RW response. No similar cases have been reported of differing RW response resulting in variation of wood composition in angiosperms. Surprisingly, only half of the genotypes tested here had a significantly reduced amount of lignin within the stems, a finding which has relevance to the later associations with glucan accessibility. The lignin and glucan contents were *not* tightly coupled in a mutually compensatory relationship and the changes in lignin content were less pronounced (by mass) than shifts in glucan content.

No significant correlations between glucan content, or lignin content, and glucan accessibility were observed in either the control or RW induced pot-grown trees. This is consistent with previous findings for SRC willows [24] and other recently published work [22,34,35], suggesting the principal factor in glucan accessibility is beyond straightforward composition alone. There was a general trend of RW induction resulting in increased glucan accessibility as well as (when combined with trends of increased glucan content) increased final glucose yields. The genotype ‘K8-428’ had a significantly increased final glucose yield (per gram of DM) due to its increased glucan content, but without any change to its glucan accessibility. Genotypes such as ‘Resolution’ were greatly improved both in cell wall composition and accessibility whereas the genotype ‘K8-088’ showed no significant change to any assessed cell wall trait. Cell wall accessibility did change significantly in the genotypes ‘Asgerd’ and ‘Endurance’ without any appreciable alteration to lignin content. This finding, in conjunction with only relatively small changes observed in lignin content for only half of the genotypes, provides further evidence of a relatively small role for lignin *content* alone in willow glucan accessibility.

Most importantly, not only do these general trends reaffirm how a RW response can be potentially beneficial to final biofuel yields but they also show that this RW response is a trait that varies between genotypes. The ability to dissect the contributions to glucose yield, being either glucan *amount* or glucan *accessibility*, is crucial in separating beneficial biofuel traits and therefore, will be essential in governing genotype selection.

Studying the influence of RW is made difficult by the fact that the amount of RW in a tree cannot currently be assessed accurately (Figures 1B and 2D). The histological analysis of two genotypes, differing in their accessibility traits (‘Shrubby’ and ‘K8-428’), illustrated the difficulties associated with relying on a single aspect of RW. The presence or absence of G-fibres has previously been described as defining the beneficial trait of increased glucan accessibility [19,20], yet the images in Figure 2D indicate that G-fibre abundance alone does not provide a reliable indicator of glucan accessibility of the whole tree. In addition, it is reported that under RW inducing conditions not all angiosperm species produce TW with G-fibres [36,37]. Another observation of note for ‘K8-428’ is the near absence of G-fibres in the control section and their presence in the induced section, yet there is no significant effect on glucan accessibility at the whole tree level. It was for this reason that assessment of what we have defined as a RW response for these studies focused on a more holistic characterisation indicated by glucose release at the whole tree level and not on the quantification of G-fibre abundance alone. It will be highly desirable to develop further independent and objective measures of the overall RW response, including accurate quantification of the extent and nature of TW, OW and NW, that can be used to further validate the findings of the present work.

### Variation in RW response contributes to mature field-grown phenotype

The most significant finding of the present research was that the variation in glucan accessibility of juvenile RW induced pot-grown trees of different genotypes (leant at 45°) was able to account for a very large proportion of the variation in glucan accessibility of mature field-grown trees of the same genotypes (in which RW had not been artificially induced). Conversely, the glucan accessibility of pot-grown control trees for these same genotypes (grown without RW induction) did not significantly account for any of the variation in glucan accessibility in the mature field-grown trees. These results provide a clear demonstration that, in this case, genotypic variation in RW response was an important trait in the field that can lead to stem biomass with improved glucan accessibility — a finding that is highly valuable for improvement of downstream processing for biofuels. Detailed characterisation of the field-grown willow trees had not revealed previously any elements of composition or tree architecture which could describe to any degree of significance the variation observed in glucan accessibility between different genotypes [24]. Indeed, the lack of straightforward associations with glucan accessibility was one of the factors that led us to investigate the RW response over a range of genotypes using the pot trial approach.

It should be noted that the growth facility used for the pot trial in this study had more air movement than that of normal greenhouses, with the specific intention of more closely mimicking field conditions. Stem wood from early developmental stages, such as that which occurs during the first year of establishment after planting or in greenhouse grown material, would be expected to be somewhat distinct from later growth stages which occur over many years before harvest. Such differences in composition and sugar release between juvenile and mature wood have previously been reported in poplar [38-40]. It is therefore a particularly intriguing aspect of the present work that a clear relationship was observed between the glucose release found in juvenile, RW induced trees and the equivalent mature tree phenotypes in the field. This finding may present a route to investigating the basis of RW induction in model, short-term, pot-grown systems that can reflect the expected glucan release phenotype of mature field grown trees.

We believe that these results reveal that the RW response is a primary cause of the variation in cell wall glucan accessibility seen in field-grown SRC willow. This led us to hypothesise that trees grown in higher RW inducing field conditions should have higher cell wall glucan accessibility. The availability of biomass samples from a potentially higher RW inducing field environment at Orkney provided an opportunity to test this.

### Impact of potentially higher reaction wood inducing conditions on the mature field-grown phenotype

The extensive influence of wind on numerous elements of tree development has been investigated in detail and is well reviewed [41]. Thigmomorphogenisis is the impact of mechanical perturbation (including wind-induced) on tree development [42,43]. Wind-induced thigmomorphogensis has been studied recently in poplar [44,45] and revealed, in general, to induce a more compact growth form comprising shorter and denser stems. These are traits that could also be associated with RW, but specific effects on cell wall development have been less well documented. If the prevailing wind pressure is sufficiently asymmetrical and consistent then stems could potentially be displaced from the vertical long enough to induce a gravitropic response (traditionally considered as distinct from thigmomorphogenesis) and more certainly lead to significant RW formation.

If glucan accessibility is strongly linked to RW induction (as the pot trial findings suggest) then increased glucose release yields in mature trees at the Orkney site would provide supportive evidence. Our results demonstrated substantial increases in glucan accessibility for all genotypes and increased glucan content for all but two of the genotypes at the Orkney site when compared with the RRes site. Whilst it cannot as yet be categorically established that these whole tree level changes in cell wall composition and glucan accessibility are due exclusively to the RW response of these genotypes, the results from the Orkney site are supportive of this contention. These observed increases in glucan yields from fully mature trees under a standard enzymatic saccharification in the laboratory would also represent major increases in maximum glucose yields per ton of biomass at the practical scale.

### The importance of low and high reaction wood inducing conditions on biofuel potential

**Figure 5:**
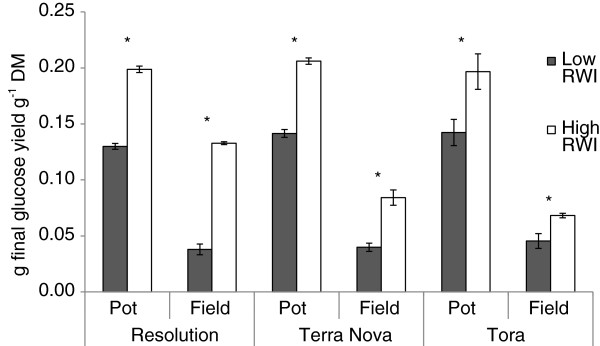
**Comparison of enzymatic saccharification yields of the three genotypes present in the pot trial and the Rothamsted Research** (**RRes**) **and Orkney field sites.** Yields are presented as grams of glucose release per gram of biomass and so encompass variation in both glucan content and glucan accessibility. Low reaction wood inducing (RWI) conditions = control trees (pot) and RRes site (field). High RWI conditions = RW induced trees (pot) and Orkney site (field). Error bars represent standard error (n = 3 trees). * Significant difference (*t*-test, p < 0.05).

Only three genotypes were included in the pot trial and in both field trials (‘Resolution’, ‘Terra Nova’ and ‘Tora’). When these are compared directly a clear pattern emerges in which higher RW inducing conditions substantially increase both glucan content and glucan accessibility. The relative changes in the *amount* of glucan and the *accessibility* of that glucan result in a large influence on the final yields of glucose per amount of biomass. For example, final glucose yields more than quadruple from 0.03 to 0.13 g per gram of DM in the field-grown genotype ‘Resolution’ under the higher RW inducing conditions (Figure 5).

These yield increases, per unit mass of biomass, in genotypes with a strong positive RW response could have radical effects on ethanol yields attainable from biomass without a pretreatment step, and potentially large effects on the pretreatment process (such as reduced severity/inhibitor production [24]). A recent life cycle assessment (LCA) of the environmental and economic sustainability of willow in the UK performed by Stephenson et al. [46] proposed a minimum 70% conversion of biomass glucan to ethanol in an optimised process system including dilute acid pretreatment (or 0.3 g of glucose per gram of DM, assuming 42.5% glucan content). Whilst the maximum final yields in the present work still fall short of those needed to completely avoid a pretreatment step, the substantial increases achieved here via this RW response alone (and without deliberate selective breeding for its enhancement) still represent an important advance in our understanding of desirable biomass traits for improving biofuel potential.

## Conclusions

Our findings provide evidence for a new concept concerning variation in the recalcitrance to enzymatic saccharification of the stem biomass of different willow genotypes (and potentially other angiosperms), namely that genotypic differences in the ability to produce a response to RW inducing conditions (the ‘RW response’) may be a primary determinant of the variation observed in cell wall glucan accessibility. It remains to be established whether the substantial differences in glucan accessibility found in this work are caused by variation in the *amount* and/or the *type* of either TW or OW. When these findings concerning the substantial contribution of the RW response to glucan accessibility were investigated in mature, field-grown trees at a potentially high RW inducing environment in Orkney, all of the genotypes were found to have greatly improved glucose release yields (up to five fold) when compared with counterparts grown under more sheltered conditions. The scope for such improved biomass to reduce the severity of lignocellulosic biofuel process chains is significant and is at the heart of achieving sustainable production of liquid transport fuels from lignocellulosic feedstocks. The identification of the importance of this RW response trait in willows (and potentially other angiosperms), offers a further target for selective breeding programs aimed at increasing glucose yields per hectare of land, decreasing costs of biofuel process chains and increasing biofuel sustainability. Furthermore, as the RW response resides within the confines of natural metabolic plasticity, it represents a cell wall alteration mechanism likely to produce a mature phenotype without loss of cell wall integrity and thereby provides an attractive target for genetic modification.

## Materials and methods

### Plant material and experimental set up

Cuttings (200 mm length by 10–15 mm diameter) made from 8 willow genotypes (Table 1), grown in a RRes reference population, selected on the basis of cell wall compositional and glucan accessibility traits [24], were planted in 12 l pots with 10 l of growing medium consisting of ^1^/_3_ vermiculite, ^1^/_3_ sharp sand and ^1^/_3_ John Innes No.2 compost, by volume. All cuttings were grown in a controlled environment with a 16 h (23°C) day cycle and an 8 h (18°C) night cycle for 42 days. Buds were limited to three per cutting. After 42 days, all stems were tied to a supporting bamboo cane at regular intervals. RW was induced by tipping the pots and stems at a 45° angle to the horizontal. For each genotype 3 trees were tipped and 3 control trees remained vertical. All trees were checked at regular intervals to ensure all stem growth was maintained in the correct growth orientation i.e. 45° or vertical, and to minimise the impact of the gravitropic response (in the tipped trees) of the apical meristem returning to vertical growth. All trees were left for another 42 days before being harvested.

**Table 1:** Species or pedigree of all 13 genotypes used in this study

**Genotype**/ **cultivar**	**Pedigree**
**Asgerd**	*S*. *viminalis* L. ‘Astrid’x (*S*. *schwerinii* Wolf x *S*. *viminalis* ‘Bjorn’)
**Terra Nova**	*S*. *triandra* L. *x* (*S*. *viminalis* LA940140 *x S*. *miyabeana* L. ‘Shrubby’)
**Shrubby**	*S*. *miyabeana* L.
**Tora**	*S*. *schwerinii* L79069 x *S*. *viminalis* ‘Orm’
**Endurance**	*S*. *rehderiana* Schneid. x *S*. *dasyclados* Skv. 77056
**Sven**	*S*. *viminalis* ‘Jorrun’ x (*S*. *schwerinii* x *S*. *viminalis* ‘Bjorn’)
**Ashton Stott**	*S*. *viminalis* ‘Bowles Hybrid’ x *S*. *burjatica* Nasarov ‘Korso’
**Tordis**	(*S*. *schwerinii* x *S*. *viminalis* ‘Tora’) x *S*. *viminalis* ‘Ulv’
**Discovery**	*S*. *schwerinii* x (*S*. *schwerinii* x *S*. *viminalis* ‘Bjorn’)
**Torhild**	(*S*. *schwerinii* x *S*.*viminalis* ‘Tora’) x *S*. *viminalis* ‘Orm’
**Resolution**	(*S*. *viminalis*. x (*S*. *viminalis*. x *S*. *schwerinii* SW930812)) x (*S*. *viminalis*. x (*S*. *viminalis*. x *S*. *schwerinii* ‘Quest’))
**K8**-**428**	(*S*. *viminalis* ‘Astrid’ x (*S*. *viminalis* ‘Astrid’ x (*S*. *schwer*. x *S*. *vim*. SW930984))S3) x (*S*. *viminalis* ‘Astrid’ x (*S*. *viminalis* ‘Astrid’ x (*S*. *schwer*. x *S*. *vim*. SW930984)) R13)
**K8**-**088**	(*S*. *viminalis* ‘Astrid’ x (*S*. *viminalis* ‘Astrid’ x (*S*. *schwer*. x *S*. *vim*. SW930984))S3) x (*S*. *viminalis* ‘Astrid’ x (*S*. *viminalis* ‘Astrid’ x (*S*. *schwer*. x *S*. *vim*. SW930984)) R13)

The mature field population at RRes is described in Ray et al. [24] and the stems at harvest were 3 years old. The mature field population in Orkney (a group of islands located north of the Scottish mainland, site at 58° 59^′^ N, 2° 59^′^ W) was established in 2007, cutback early in 2008 and the stems harvested at the end of the first harvest cycle in January 2012 when 4 years old.

### Sample harvesting & processing

All six pot trees per genotype (3 control + 3 tipped) were cut down and all the leaves removed, harvesting all the above-ground stem biomass. All stems were harvested from each tree and weighed to determine DM biomass yields. The stems (bark on) were cut into smaller segments, split longitudinally and left to air dry at room temperature. All the stems from a tree were collectively milled and sieved to a defined particle size of between 850 and 180 μm using a Retsch® SM 2000 cutter mill, in accordance with Hames et al. [47]. Moisture contents were determined by oven drying sub samples at 105°C and calculated as a percentage of DM. This air dried, milled biomass was used in all of the subsequent analysis. The field grown trees from RRes were harvested as described by Ray et al. [24]. For the mature field grown trees from Orkney, all the above-ground stem biomass was chipped for each tree before being milled and sieved as above.

Samples were collected from a stem at the mid-point of each tree used in the present research for sectioning (2 cm or 5 cm long for the pot and field trials respectively). The transverse sections of these samples were made (at a thickness of ~20 μm) using a Reichert sledge microtome. Staining was performed to visually assess the presence of G-fibres by using either 1% Chlorazol Black E in methoxyethanol [21] alone or 1% aqueous Safranin O and 1% Chlorazol Black E in methoxyethanol, and were permanently mounted in DPX. All samples both pot and field were found to contain G-fibres to some degree.

### Compositional analysis

Milled biomass was extracted with 95% ethanol prior to compositional analysis according to Sluiter et al. [48], using a Dionex® Accelerated Solvent Extractor (ASE200). Extracted biomass was analysed for structural carbohydrates and lignin in accordance with Sluiter et al. [49]. All sugars were assessed using a Bio-Rad Aminex HPX-87P column at 80°C with a flow rate of 0.6 mL min^−1^ water mobile phase on an Agilent 1200 series HPLC.

### Enzymatic saccharification

Saccharification assays were carried out for 7 days following the procedure of Selig et al. [50] with a 1:1 ratio of two commercially available cellulase mixtures: Celluclast 1.5 L and Novozyme 188 (cellobiase from *Aspergillus niger*) (Sigma, Gillingham, UK) at 60 FPU/g glucan. Glucose release per gram of glucan includes an anhydro correction factor, as outlined in the procedure [50], to account for the addition of a water molecule upon depolymerisation. Final glucose yields per gram of DM do not contain an anhydro correction factor as their purpose is to present actual glucose yield outputs, hence final glucose yields should not be used to reflect the residue DM from the process. Free monomeric glucose within the biomass was assessed and subtracted from all glucose release values. Maximum starch concentrations in willow stems have been reported as < 0.6% DM [51] so will not noticeably impact glucose release yields. All the stem samples were assayed for saccharification with the bark included. Glucose concentrations were assessed by HPLC as described above.

### Phenotype terminology

We ascribe the effects observed here to a RW response and we have deliberately avoided inferences or implications that the effects derive from TW alone. All the components of RW, including the proportions and type of TW, OW and NW, may contribute to the aggregate extent of RW. In the current absence of any reliable and universally accepted quantification mechanism for RW, we have been careful to avoid categorical interpretation of our results in terms of direct linkage to any specific component such as TW. However, we do believe that the present results demonstrate clearly that, under conditions known to induce RW, specific (but not all) willow genotypes clearly develop an interesting and valuable low recalcitrance phenotype.

### Statistical analysis

Genstat® was used to analyse the glucose release data from each genotype for correlation coefficients between treatments. The correlation coefficients and their significance (p-values) are given. ANOVA was used to determine statistical differences between genotypes for each trait. Student’s *T*-test was used to determine statistical significance of treatments within a genotype.

## Abbreviations

DM: Dry matter; NW: Normal wood; OW: Opposite wood; RRes: Rothamsted Research; RW: Reaction wood; SRC: Short rotation coppice.

## Competing interests

The authors declare that they have no competing interests.

## Authors’ contributions

NJBB, MJR and IS designed the study. NJBB and MJR performed the experiments, interpreted the data and drafted the manuscript. AK is overall leader of the BSBEC BioMaSS project and RJM leads the sub programme of which this work is a part. IS, PM and AK designed and established the field trials. All authors conceived the study, commented on the results and contributed to the manuscript.

## Supplementary Material

Additional file 1 **Table S1.** Biomass composition. Compositional values of raw willow biomass are presented here as genotype means as a percentage of dry matter (DM) and biomass (yield) as DM grams of stem. Standard error is displayed in brackets (n = 3 trees). Ash content for the genotypes was uniformly small (<1% DM) and is not presented here but is included in the final mass closure values. Compositional data for these genotypes grown at the RRes field site has previously been published in Ray *et al*[24]. Reaction wood (RW). Click here for file
