# Supplementary material for: Reaction wood – a key cause of variation in cell wall recalcitrance in willow
Source: Biotechnol Biofuels. 2012 Nov 22;5:83. doi: 10.1186/1754-6834-5-83 (PMC3541151; doi:10.1186/1754-6834-5-83)
Supplement: Additional file 1 — Table S1. Biomass composition. Compositional values of raw willow biomass are presented here as genotype means as a percentage of dry matter (DM) and biomass (yield) as DM grams of stem. Standard error is displayed in brackets (n = 3 trees). Ash content for the genotypes was uniformly small (<1% DM) and is not presented here but is included in the final mass closure values. Compositional data for these genotypes grown at the RRes field site has previously been published in Ray et al[24]. Reaction wood (RW). [file 1754-6834-5-83-S1.docx]

**Supplementary Table 1 Biomass composition**

Compositional values of raw willow biomass are presented here as genotype means as a percentage of dry matter (DM) and biomass (yield) as DM grams of stem. Standard error is displayed in brackets (n = 3 trees). Ash content for the genotypes was uniformly small (< 1 % DM) and is not presented here but is included in the final mass closure values. Compositional data for these genotypes grown at the RRes field site has previously been published in Ray *et al* [23]. Reaction wood (RW).

| **Genotype** | **Trial/Treatment** | **Extractives** | **Glucan** | **Xylan** | **Galactan** | **Arabinan** | **Mannan** | **Lignin** | **Mass closure** | **Biomass** |
| --- | --- | --- | --- | --- | --- | --- | --- | --- | --- | --- |
| **K8-428** | Pot-grown, control | 7.94 (0.18) | 38.79 (0.17) | 10.51 (0.46) | 1.92 (0.04) | 2.45 (0.07) | 3.91 (0.15) | 24.31 (0.20) | 10.16 (0.56) | 36.01 (2.19) |
| **Asgerd** | Pot-grown, control | 8.18 (0.45) | 38.86 (1.79) | 10.18 (0.05) | 1.69 (0.08) | 2.20 (0.04) | 3.82 (0.08) | 24.43 (0.71) | 10.65 (2.36) | 44.44 (2.88) |
| **Terra Nova** | Pot-grown, control | 10.41 (0.22) | 37.38 (0.22) | 11.39 (0.23) | 2.09 (0.07) | 3.23 (0.05) | 3.47 (0.13) | 25.13 (0.09) | 6.90 (0.27) | 18.52 (1.62) |
| **Resolution** | Pot-grown, control | 8.56 (0.25) | 37.88 (0.35) | 12.57 (0.13) | 1.95 (0.05) | 2.49 (0.01) | 4.07 (0.05) | 25.23 (0.50) | 7.26 (0.25) | 38.10 (1.41) |
| **Shrubby** | Pot-grown, control | 9.71 (0.14) | 39.41 (0.75) | 12.60 (0.32) | 2.12 (0.06) | 3.28 (0.10) | 3.18 (0.03) | 24.34 (0.37) | 5.36 (0.22) | 28.23 (1.50) |
| **K8-088** | Pot-grown, control | 8.84 (0.99) | 42.66 (0.31) | 12.49 (0.03) | 2.61 (0.09) | 3.01 (0.45) | 4.09 (0.09) | 23.86 (0.84) | 2.43 (1.88) | 29.78 (1.93) |
| **Tora** | Pot-grown, control | 8.75 (0.32) | 39.42 (0.71) | 12.14 (0.44) | 1.41 (0.18) | 3.33 (0.08) | 3.20 (0.09) | 25.54 (0.15) | 6.21 (0.56) | 31.80 (1.98) |
| **Endurance** | Pot-grown, control | 11.26 (0.60) | 39.60 (0.72) | 12.00 (0.14) | 1.70 (0.10) | 4.26 (0.20) | 4.72 (0.81) | 22.09 (0.21) | 4.37 (1.40) | 21.83 (1.94) |
| **K8-428** | Pot-grown, RW induced | 8.02 (0.23) | 41.86 (1.01) | 11.20 (0.22) | 2.07 (0.05) | 2.63 (0.14) | 4.05 (0.12) | 22.92 (0.65) | 7.24 (0.28) | 31.24 (1.33) |
| **Asgerd** | Pot-grown, RW induced | 8.09 (0.48) | 38.44 (0.91) | 9.52 (0.11) | 1.82 (0.02) | 2.42 (0.31) | 3.88 (0.15) | 23.78 (0.71) | 12.05 (0.41) | 39.38 (1.67) |
| **Terra Nova** | Pot-grown, RW induced | 8.90 (0.39) | 42.67 (0.55) | 10.69 (0.12) | 2.28 (0.17) | 2.93 (0.09) | 3.52 (0.28) | 23.12 (0.24) | 5.90 (0.32) | 14.62 (0.09) |
| **Resolution** | Pot-grown, RW induced | 9.33 (0.27) | 42.71 (0.43) | 11.23 (0.02) | 2.30 (0.04) | 2.68 (0.04) | 4.43 (0.05) | 23.33 (0.55) | 3.99 (0.18) | 31.90 (3.36) |
| **Shrubby** | Pot-grown, RW induced | 9.20 (0.40) | 42.29 (0.50) | 10.45 (0.55) | 2.10 (0.14) | 2.74 (0.27) | 3.02 (0.13) | 22.72 (0.17) | 7.48 (0.64) | 24.67 (2.49) |
| **K8-088** | Pot-grown, RW induced | 7.95 (0.51) | 43.93 (0.73) | 12.42 (0.40) | 2.44 (0.08) | 2.70 (0.27) | 4.54 (0.17) | 22.71 (0.43) | 3.32 (0.82) | 26.97 (2.86) |
| **Tora** | Pot-grown, RW induced | 8.53 (0.07) | 43.27 (0.55) | 11.45 (0.42) | 1.04 (0.06) | 3.74 (0.38) | 4.13 (0.34) | 23.18 (0.50) | 4.65 (1.08) | 32.13 (0.81) |
| **Endurance** | Pot-grown, RW induced | 10.11 (0.13) | 43.01 (1.09) | 11.38 (0.13) | 1.37 (0.18) | 3.62 (0.24) | 4.03 (0.80) | 21.65 (0.56) | 4.82 (1.28) | 22.40 (0.76) |
| **Sven** | Field-grown, Orkney | 5.30 (0.22) | 43.43 (0.37) | 14.53 (0.49) | 2.62 (0.09) | 1.89 (0.07) | 1.90 (0.10) | 26.28 (0.54) | 3.96 (1.03) | N/A |
| **Ashton Stott** | Field-grown, Orkney | 6.97 (0.42) | 44.13 (0.60) | 14.49 (0.76) | 2.67 (0.03) | 2.04 (0.12) | 1.66 (0.02) | 26.04 (0.61) | 2.00 (1.77) | N/A |
| **Tordis** | Field-grown, Orkney | 6.93 (0.52) | 38.70 (0.58) | 14.72 (0.36) | 2.61 (0.01) | 2.04 (0.03) | 1.60 (0.06) | 28.74 (0.29) | 4.57 (0.23) | N/A |
| **Discovery** | Field-grown, Orkney | 5.62 (0.47) | 41.81 (1.21) | 15.72 (0.57) | 2.64 (0.05) | 1.86 (0.07) | 1.36 (0.02) | 28.73 (0.45) | 2.26 (0.97) | N/A |
| **Resolution** | Field-grown, Orkney | 5.85 (0.27) | 42.69 (0.65) | 15.20 (0.28) | 2.95 (0.13) | 2.74 (0.08) | 2.52 (0.03) | 26.37 (0.05) | 1.68 (0.53) | N/A |
| **Torhild** | Field-grown, Orkney | 3.84 (0.74) | 44.17 (1.40) | 16.21 (0.31) | 3.08 (0.10) | 3.02 (0.22) | 2.44 (0.11) | 27.09 (0.97) | 0.14 (0.65) | N/A |
| **Terra Nova** | Field-grown, Orkney | 8.15 (0.14) | 39.72 (0.45) | 15.37 (0.27) | 2.99 (0.17) | 3.67 (0.18) | 2.80 (0.22) | 28.63 (0.80) | -1.34 (0.49) | N/A |
| **Tora** | Field-grown, Orkney | 4.83 (0.32) | 39.58 (0.33) | 16.73 (0.55) | 2.91 (0.18) | 3.32 (0.22) | 2.71 (0.22) | 27.78 (0.53) | 2.15 (0.32) | N/A |
